# Cyclophilin D knockout mice do not accumulate succinate during cardiac ischemia

**DOI:** 10.1016/j.yjmcc.2022.09.006

**Published:** 2022-10-06

**Authors:** Hiran A. Prag, Duvaraka Kula-Alwar, Paolo Bernardi, Fabio Di Lisa, Michael P. Murphy, Thomas Krieg

**Affiliations:** a Department of Medicine, University of Cambridge, Cambridge Biomedical Campus, Hills Road, Cambridge, United Kingdom, CB2 0QQ; b MRC Mitochondrial Biology Unit, University of Cambridge, Cambridge Biomedical Campus, Hills Road, Cambridge, United Kingdom, CB2 0XY; c Department of Biomedical Sciences, University of Padova, 35131, Padova, Italy; d Neuroscience Institute, National Research Council of Italy (CNR), 35131, Padova, Italy

**Keywords:** Ischemia/reperfusion injury, Succinate, Mitochondrial permeability transition pore, Cyclophilin D, Cyclosporin A, Reactive oxygen species

Prof Rong Tian

Editor-in-Chief

JMCC

Dear Prof Tian

23 September 2022

Re: JMCC15068: *Cyclophilin D knockout mice do not accumulate succinate during cardiac ischemia*

Thank you for your decision letter about this submission. We are delighted that you are prepared to consider a revised version of this paper. Below, we have responded to the specific comments of the reviewers and added new data, changed the text accordingly and reduced the number of references. We hope that the revised paper is now acceptable for publication.

Yours sincerely

Thomas Krieg on behalf of all the authors

## Response to Reviewers’ Comments

### Reviewer #2

#### Comment

The revised letter by Prag et al is much improved with regards to clarity and the scope of the conclusions that the authors draw. The authors have satisfactorily addressed my major comments that do not require further experimentation. The only remaining edits I would suggest are in the interest of helping the reader integrate this new finding on succinate accumulation with the prior functional studies by this group.

#### Response

We thank the reviewer for their positive comments on the changes made

#### Comment

**Fig. 1:**
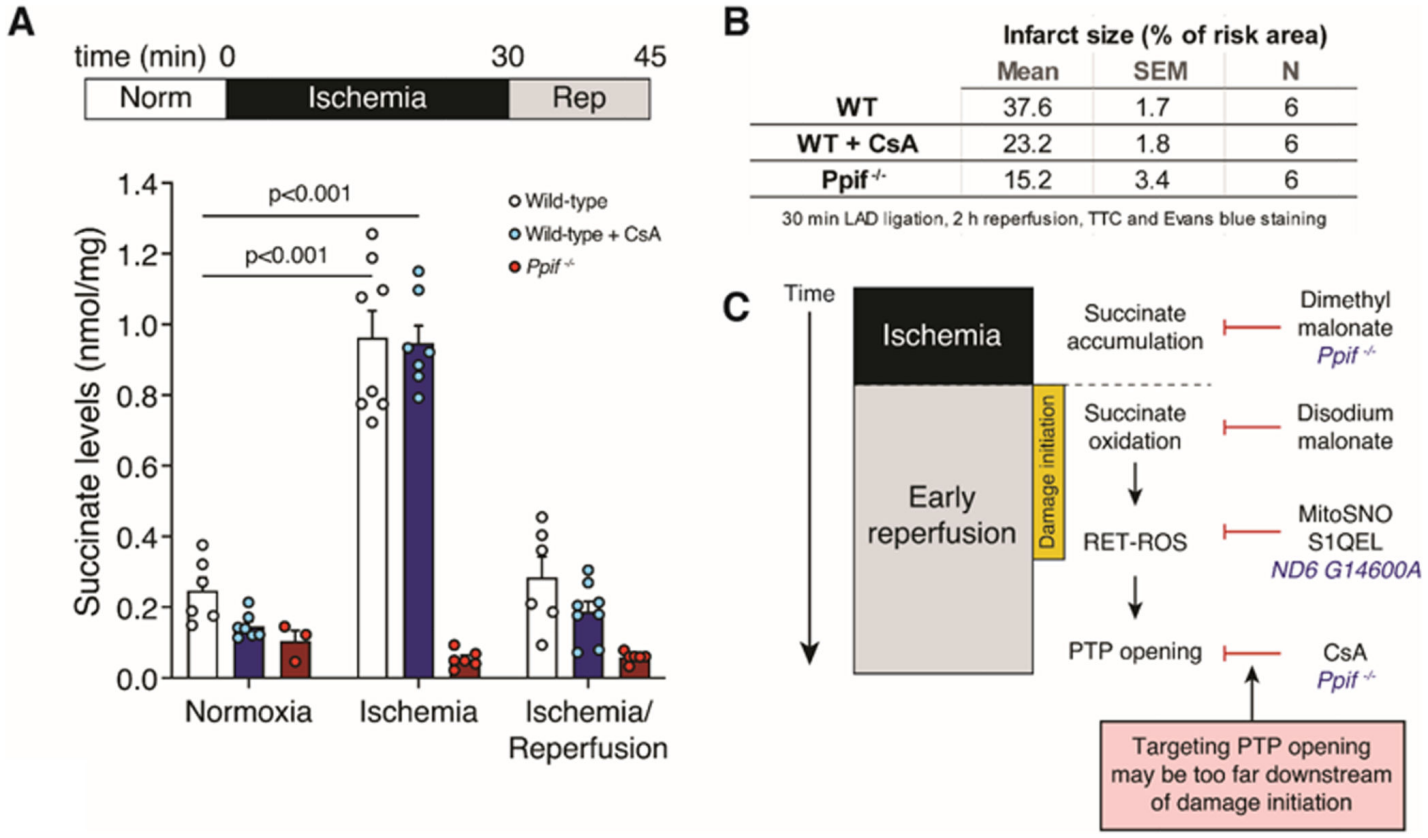
Succinate does not accumulate in the ischemic *Ppif*^*-/-*^ mouse heart. (A) *Ppif*^*-/-*^ or wild-type (WT) C57BL/6J mice were subjected to *in vivo* LAD ligation for 30 min, or 30 min ischemia followed by 15 min reperfusion, before rapidly clamp freezing heart tissue, and succinate levels measured by mass spectrometry. CsA (10 mg/kg total) was infused i.v. during normoxia. (Mean ± S.E.M., n = 3-8 biological replicates. Statistics: two-way ANOVA with Dunnett’s post-hoc test versus wild-type normoxia). (B) Infarct size after 30 min ischemia and 2 h reperfusion under the conditions described in (A). (C) Timeline of ischemia and early reperfusion, with potential pharmacological or genetic interventions.

1) (original comment #2) Fig. 1A: it would be helpful to provide the data showing the extent of I/R injury in all mouse groups tested alongside their succinate levels.

#### Response

While infarct size cannot be measured in the same samples as succinate, we have now added a summary of measured infarcts to figure 1 as a new panel B.

#### Comment

2) Line 36: here, please cite the original paper demonstrating protection of Ppif-/- mice from IRI containing these data to help direct the reader to these functional measurements.

#### Response

We have now included the summary of infarct data under the different conditions. We have not published the protection from Ppif^-/-^ previously, as many others have, and have cited the original paper here too (Page 2, line 35-36).

## Dear Editor

A key driver of cardiac ischemia/reperfusion injury (IRI) and target for cardioprotection is the mitochondrial permeability transition pore (PTP). Persistent opening of the PTP leads to depolarization and increased permeability of the mitochondrial inner membrane followed by swelling and rupture, culminating in cell death [1]. The molecular characterization of the PTP remains contentious, however, cyclophilin D (CyD; a mitochondrial matrix peptidyl prolyl isomerase encoded by *Ppif*) is an important PTP modulator. Much of the evidence for the pathological role of the PTP in IRI comes from *Ppif*^*-/-*^ mice which lack CyD [2]. Isolated mitochondria from these mice are highly resistant to PTP-induction and their hearts are profoundly protected against IRI *in vivo*. Based in part on observations from *Ppif*^*-/-*^ mice, CyD has become a major target for the treatment of IRI, most notably through its potent inhibition by cyclosporin A (CsA). Blocking the PTP with CsA showed promise both *in vitro* and in pre-clinical animal models, although translation to patients was disappointing [3].

Recent work has shown that the mitochondrial metabolite succinate is key to causing the oxidative damage that triggers the PTP opening and drives IRI [4]. Succinate dramatically accumulates in ischemic heart tissue, and upon reperfusion, it is rapidly oxidized back to baseline levels within approximately one minute of reperfusion by succinate dehydrogenase (SDH). This rapid succinate oxidation drives the production of the reactive oxygen species (ROS) superoxide by reverse electron transport (RET) through mitochondrial complex I. The burst of RET-ROS upon reperfusion initiates the damage that leads to PTP opening and the subsequent cell death seen in IRI [4]. Cardioprotection in a RET-ROS-null mouse model, or by inhibition of succinate oxidation at SDH by the competitive inhibitor malonate support the key role of succinate-derived RET-ROS in initiating the oxidative damage that subsequently induces the PTP and leads to cell death and IRI [4–6]. This pathological sequencing of RET-driven superoxide production leading to PTP induction was supported by the Brookes lab [7], who showed that PTP inhibition with CsA had no impact on the burst of RET-ROS upon reperfusion, whereas in contrast SDH inhibition or selective blockage of complex I prevented RET-ROS at reperfusion (Fig. 1).

Here, we explored the role of succinate accumulation and oxidation during IRI in *Ppif*^*-/-*^ mice (Fig. 1). As expected, succinate accumulated extensively in the ischemic heart tissue of WT mice. Treatment with CsA protected WT mice against cardiac IRI without affecting succinate accumulation during ischemia, or its oxidation during reperfusion (Fig. 1). Corroborating previous reports [2], *Ppif*^*-/-*^ mice were protected against IRI in our hands (Fig. 1). However, to our surprise, we found that when their hearts were subjected to left anterior descending coronary artery (LAD) ligation, there was no increase of succinate in the ischemic tissue (Fig. 1). This is a striking result as succinate accumulation is a conserved response in ischemic cardiac tissue in mice, rabbits, pigs and humans [8]. To our knowledge, this is the only mouse model that fails to accumulate succinate during cardiac ischemia.

These data corroborate and extend the previous findings of perturbed metabolism in *Ppif*^*-/-*^ mice by the Molkentin and Murphy groups [9]. Why succinate does not accumulate during ischemia in these mice is unclear, but as acute inhibition of CyD with CsA does not affect the accumulation or oxidation of succinate, the metabolic shift in the *Ppif*^*-/-*^ mice is a long-term adaptation to chronic lack of CyD. The nature of this adaptation is beyond the scope of this work, but *Ppif*^*-/-*^ mice have reduced metabolic flexibility, favoring glucose consumption over fatty acid oxidation, along with increased mitochondrial NADH dehydrogenase activity due to elevated matrix calcium levels. Furthermore, mitochondrial proteome changes in *Ppif*^*-/-*^ mice may be implicated in the lack of succinate accumulation, such as through reduced levels of SDH subunits [10].

Succinate accumulation and oxidation is a key driver of IRI. Our findings show that the metabolic adaptation from chronic lack of CyD function in *Ppif*^*-/-*^ mice prevents succinate accumulation during ischemia and its subsequent oxidation upon reperfusion. This contrasts with the current consensus that *Ppif*^*-/-*^ mice are solely protected against cardiac IRI by preventing the PTP opening that occurs downstream of the oxidative damage that occurs upon reperfusion. Instead, our data suggest that the lack of damage during IRI in *Ppif*^*-/-*^ mice may be because *Ppif*^*-/-*^ mice do not accumulate succinate during ischemia and consequently are lacking a key driver of IRI, as well as being unable to undergo PTP opening (Fig. 1) [6].
